# Associations between Child and Family Level Correlates and Behavioural Patterns in School-Aged Children

**DOI:** 10.3390/children8111023

**Published:** 2021-11-07

**Authors:** Ninoshka J. D’Souza, Miaobing Zheng, Gavin Abbott, Sandrine Lioret, Kylie D. Hesketh

**Affiliations:** 1 Institute for Physical Activity and Nutrition, School of Exercise and Nutrition Sciences, Deakin University, Melbourne, VIC 3125, Australia; j.zheng@deakin.edu.au (M.Z.); gavin.abbott@deakin.edu.au (G.A.); kylie.hesketh@deakin.edu.au (K.D.H.); 2 Research Center in Epidemiology and Biostatistics (CRESS), Université de Paris, INSERM, INRAE, 75004 Paris, France; sandrine.lioret@inserm.fr

**Keywords:** diet, physical activity, sedentary behaviour, sleep, children, correlates, family, behavioural patterns

## Abstract

Identifying correlates of behavioural patterns are important to target population sub-groups at increased health risk. The aim was to investigate correlates of behavioural patterns comprising four behavioural domains in children. Data were from the HAPPY study when children were 6–8 years (n = 335) and 9–11 years (n = 339). Parents reported correlate and behavioural data (dietary intake, physical activity, sedentary behaviour, and sleep). Behavioural data were additionally captured using accelerometers. Latent profile analysis was used to derive patterns. Patterns were identified as healthy, unhealthy, and mixed at both time points. Multinomial logistic regression tested for associations. Girls were more likely to display healthy patterns at 6–8 years and display unhealthy and mixed patterns at 9–11 years than boys, compared to other patterns at the corresponding ages. Increased risk of displaying the unhealthy pattern with higher age was observed at both timepoints. At 9–11 years, higher parental working hours were associated with lower risk of displaying mixed patterns compared to the healthy pattern. Associations observed revealed girls and older children to be at risk for unhealthy patterns, warranting customisation of health efforts to these groups. The number of behaviours included when deriving patterns and the individual behaviours that dominate each pattern appear to be drivers of the associations for child level, but not for family level, correlates.

## 1. Introduction

Dietary intake, physical activity, sedentary behaviour, and sleep are health behaviours that have significant impact on children’s growth, wellbeing, and health [1,2]. These behaviours influence many childhood outcomes (e.g., overweight and obesity) and are key modifiable health behaviours in health promotion efforts [3,4]. These behaviours are influenced by child, family, and environmental level factors as established by the ecological framework [5,6]. For example, evidence for family level factors suggests that children from lower socio-economic backgrounds tend to display less healthy diets [7], lower physical activity levels [8], lower sleep duration [9], and higher levels of sedentary behaviour [10].

In recent years, the use of multivariate data-driven methods to examine the integrated influence of health behaviours as behavioural patterns has become increasingly popular [11,12]. Given associations between child, family, and environmental level correlates and individual health behaviours are established [13,14,15], it is logical to posit these correlates might also impact the behavioural patterns these individual behaviours co-occur in.

Previous reviews [12,15,16] examining associations between child and family level correlates and behavioural patterns identified most studies investigated patterns comprising 2–3 behavioural domains only (dietary intake, physical activity, and/or sedentary behaviour). Most frequently explored child and family level correlates include child sex and parental education level (as a proxy of socio-economic position (SEP)). Others include child age, parent occupation [17], and maternal working hours [18]. Older children (9–12 y) exhibited patterns with lower levels of physical activity and younger children displayed healthier patterns comparatively [15], consistent with the evidence for individual behaviours. Most studies included children above nine years [12], thus evidence for younger children remains unclear.

Only six studies [11,19,20,21,22,23] have investigated potential correlates of patterns derived from four behavioural domains (diet, physical activity, sedentary behaviour, and sleep). All six studies investigated associations with child sex. Findings revealed associations by sex were not significant at 6–8 y (21), but at 9–12 y girls exhibited unhealthy and mixed patterns [11,22,23]. Two studies [19,20] derived patterns separately for boys and girls. One found similar patterns by sex [19], and the other [20] reported boys to be more sedentary and girls to have lower activity patterns. Within the six studies, family level correlates investigated included parental education, place of residence, and SEP. Higher maternal education showed evidence of associations with healthier patterns [23], and lower parental education showed evidence of associations with unhealthier patterns [20]. Little evidence of associations was reported between patterns and place of residence [21] or combined parent education and occupation derived socioeconomic status [11].

Other family level factors that have been shown to be correlated with individual health behaviours [24] but not yet explored in relation to behavioural patterns comprising all four behavioural domains include parent working hours, indicators of low income (e.g., possession of a health care or pension card), and family composition (parent marital status and number of siblings). Exploring these associations can help identify population sub-groups these patterns emerge in and their subsequent risks for health outcomes.

This study aimed to investigate cross-sectional associations between child and family level correlates and behavioural patterns derived from four health behaviours (diet, physical activity, sedentary behaviour, and sleep) in two age groups (6–8 and 9–11 y of age). Given that the patterns identified were different at the two time points, we explored these associations cross-sectionally. The study is novel for its inclusion of four behavioural domains in pattern derivation and investigation of understudied family level correlates.

## 2. Materials and Methods

Data of children aged 6–8 y and 9–11 y from the Healthy Active Preschool and Primary Years (HAPPY) study conducted in Melbourne, Australia, were used. The study has been discussed in detail previously [25]. In brief, it began in 2008/09 with 1002 parents of children aged 3–5 y (T1). Parents were followed up when children were 6–8 y (T2) and 9–11 y (T3) in 2011 and 2012 (77% retention rate) and 2012 and 2013 (74% retention rate), respectively. Ethics approval for this study was obtained from the Department of Education and Early Childhood Development (2011_001008), the Catholic Education Office (1714), and the Deakin University Human Research Ethics Committee (EC 291-2007). At each time point, parents provided written informed consent to participate. Dietary data were not collected at baseline, and thus children at 3–5 y were not included in these analyses.

The following measures were captured at both time points.

### 2.1. Dietary Intake

Parents reported children’s dietary data using a validated food frequency questionnaire [26] to measure intake of sweet and savoury discretionary food items (unhealthy foods) and fruit and vegetable (healthy foods) intake. The survey included only those items that showed sufficient reliability [26]. Frequency of discretionary food items consumed in the previous week was captured using a 7-point Likert scale (range: 0–6 or more times). Six sweet (peanut butter or Nutella spread, chocolate, ice-cream, pre-sugared cereals, lollies and snack bars, and bakery items) and seven savoury (cheese or cheese spreads, sausage rolls and pies, potato crisps or savoury biscuits, pizza, french fries or hot chips, processed meat or hot dogs, and takeaway food) discretionary food items, respectively, were summed and then divided by seven to obtain daily food intake. Frequency of overall fruit and vegetable intake in the past 24 h was captured using a 6-point Likert scale (range: 0–5 or more times) to obtain daily fruit and vegetable intake, respectively.

### 2.2. Physical Activity

Parents reported the time (in hours and minutes) their children spent in organised sports and outdoor play [27]. For a typical week, total weekly duration of a list of organised sports (basketball, netball, soccer, football, swimming, gymnastics, dance, cricket, and “other” sports) were individually captured. Total weekly duration for each individual organised sport was summed and divided by seven to obtain average daily organised sport duration. For a typical weekday and weekend day, time spent playing outdoors was also reported. Weekday (Monday to Friday) and weekend (Saturday to Sunday) values were multiplied by five and two, respectively, then summed and divided by seven to obtain average daily outdoor play time.

### 2.3. Sedentary Behaviour

Parents reported the time (in hours and minutes) their children spent in various sedentary behaviours across a typical week and on a typical weekend. These were collapsed into screen time (television viewing plus computer use excluding games), videogames (computer games plus handheld electronic games), and quiet play. The total time spent in the behaviours that make up the three variables, respectively, were first summed and then divided by seven to obtain daily duration in minutes. These items showed acceptable reliability [27].

Additionally, Actigraph GT1M uniaxial accelerometers (Pensacola, FL, USA) were used to measure physical activity and sedentary behaviour objectively. Accelerometers were distributed in person. Children wore them during waking hours for eight consecutive days. These were hip-worn and were removed for any water-based activities. Accelerometer data were recorded in 15 s epochs [28] and considered valid if recorded for a minimum of eight hours a day for ≥4 days, inclusive of one weekend day. Data consisting of consecutive zeros ≥10 min were defined as non-wear time. Moderate- to vigorous-intensity physical activity (MVPA) and sedentary time were classified as counts >2296 min and counts <100 min, respectively [29]. Accelerometer data were regressed on wear time to obtain residuals to adjust MVPA and sedentary time to total wear time. At T2 and T3 respectively, 534 and 522 children wore accelerometers. Valid data were, however, available for 445 (monitors lost = 3, monitors failed = 2, invalid data = 84) and 463 (monitors lost = 4, invalid data = 55) children at T2 and T3.

### 2.4. Sleep

Parents reported children’s usual sleep duration at night in hours and minutes. This item showed good reliability [27].

### 2.5. Correlates

Parents reported both child and family level correlates. Parents reported child sex and date of birth, from which age was calculated. The reporting parent’s (main caregiver - 95% mother or stepmother) education level (highest level of schooling) was captured using seven response options and grouped into university versus no university education. Time spent at work in a typical week was reported in hours and minutes per day. Marital status was captured using six response options and grouped into married/de facto and other (never married, divorced, separated, or widowed). As a measure of economic disadvantage, respondents were asked if they had a current low-income health care card or pension card. To capture information on siblings, parents reported the number and ages of other children (<18 y) living in the house, apart from the child taking part in the study. The children included in the study were grouped into four categories based on this information and their age (only child, oldest child (younger siblings), youngest child (older siblings), and middle child (both younger and older siblings)) to represent sibling type.

### 2.6. Statistical Analyses

Statistical analyses were conducted using Stata 16.0 (StataCorp, College Station, TX, USA) and Mplus 8.0 (Muthén & Muthén, Los Angeles, CA, USA). Differences between excluded and included children (at both time points) in the analyses were assessed using *t*-tests and chi-square tests, and sample demographics were assessed using descriptive statistics. Four dietary variables (sweet and savoury discretionary food intakes, fruit, and vegetables), three physical activity variables (MVPA levels (accelerometer obtained), organised sport and outdoor play duration), four sedentary behaviour variables (screen, videogame, quiet play, and sedentary time (accelerometer obtained)), and one sleep variable (sleep duration) were included in a latent profile analysis (LPA) to identify behavioural patterns. Due to the non-uniformity in the scales across behavioural variables captured, input variables were standardized prior to inclusion in LPA.

To determine the best fitting model, a range of solutions (2–10 patterns) were derived and compared according to the adjusted Lo–Mendel–Rubin (aLMR) test criteria [30]. The three-pattern model was identified to be most optimal at both time points. To interpret the estimated means of standardised behaviour scores for each pattern obtained from LPA, a cut-point of ±0.2 was utilised as a minimal differentiation point to identify behaviours as being either high or low in the patterns identified. Pattern characteristics are presented in the Supplementary Table S1. When children were 6–8 years, an “unhealthy” pattern (low fruit and vegetable and high sweet discretionary food consumption, low overall physical activity, high overall sedentary behaviour, and low sleep duration), a healthy pattern labelled “non-sedentary healthy eaters” (high fruit and vegetable and low discretionary food consumption with low screen time duration), and a mixed pattern labelled “active unhealthy eaters” (high discretionary food and low fruit and vegetable consumption, high outdoor play and MVPA, and low sedentary time) were identified. At 9–11 y, an “unhealthy” pattern (low fruit and vegetable intake, low overall physical activity, highest screen, videogame, and sedentary time, and low sleep duration), an “intermediate” pattern characterised by most behaviours being not particularly high or low compared with the sample average, and a healthy “active and non-sedentary” (high overall physical activity and low sedentary time) pattern were identified.

### 2.7. Associations between Correlates and Behavioural Patterns

Cross-sectional associations between child (age and sex) and family level (parental education, number of working hours, current marital status, health care or pension card, and sibling type) correlates and derived behavioural patterns were tested using multinomial logistic regression conducted separately for the 6–8 and 9–11 y timepoints. The healthy behavioural pattern was the reference category level (the “non-sedentary healthy eaters” and “active and non-sedentary” pattern at 6–8 and 9–11 y, respectively). Bivariable associations between each correlate and the outcome variable (behavioural patterns) were first tested, and those that showed some evidence of associations (*p* < 0.10) were included in the multivariate multinomial regression model. Potential clustering by recruitment centre was accounted for by fitting models with cluster-robust standard errors.

## 3. Results

Complete data on behavioural variables and correlates were available for 335 and 339 children at 6–8 and 9–11 y, respectively. No differences were observed for child sex, age, working hours, and sibling type between excluded and included children at either time point. However, a higher proportion of tertiary educated parents, and parents with “other” marital status were observed in the excluded sample at both time points. Lastly, parents of the excluded participants at 6–8 y were less likely to possess a health care or pension card. Sample characteristics are presented in Table 1. Children had a mean age of 7.5 y and 10.5 y at the two time points. Most parents were tertiary educated, worked at least four hours a week, did not have a health or pension card, and were currently married

Associations between child and family level correlates and behavioural patterns identified at 6–8 y are presented in Table 2. At 6–8 y, child sex and age and marital status and health care card availability showed evidence of bivariate associations and were included in the multivariate model. The multivariate model indicated that girls were 66% less likely than boys to display the “active unhealthy eaters” pattern compared to the “non-sedentary healthy eaters” pattern. Every year increase in age was associated with an estimated 53% lower risk of being in the mixed (active unhealthy eaters) pattern than the healthy (non-sedentary healthy eaters) pattern. For every additional year of age, there was an estimated 100% higher risk of children being in the unhealthy pattern than the healthy (non-sedentary healthy eaters) pattern. Little evidence of association with patterns was found for family level correlates at 6–8 y.

At 9–11 y (Table 3), child sex and age and parent working hours showed evidence of bivariable association with behavioural patterns and were included in the multivariate model. Girls were 11.63 times more likely than boys to be in the unhealthy pattern and 6.57 times more likely than boys to be in the intermediate pattern compared with the active and non-sedentary pattern. For each additional year of age there was an estimated 107% higher risk of children being in the unhealthy pattern than the active and non-sedentary pattern. Lastly, for every additional parent working hour, there was an estimated 9% lower risk of children being in the intermediate pattern compared to the active and non-sedentary pattern. Little evidence of association with behavioural patterns was found for parent education, marital status, health care or pension card possession, and sibling type at 9–11 y.

## 4. Discussion

This study investigated cross-sectional associations of behavioural patterns derived from four behavioural domains (dietary intake, physical activity, sedentary behaviour, and sleep), with child and family level correlates, in children aged 6–8 and 9–11 y. At both time points, child age and sex displayed evidence of associations with patterns. Higher number of parental working hours was associated with lower risk of displaying the intermediate pattern than the healthy pattern at 9–11 y. Little evidence of associations were observed for other family level correlates. Girls were less likely to display mixed patterns at 6–8 y and were more likely to display unhealthy and mixed patterns at 9–11y, than boys. Higher risk of exhibiting unhealthy patterns with increasing age was evident at both time points. The findings suggest that for child level correlates, evidence for associations with patterns is similar to what is reported for individual behaviours, but for family level correlates associations may be less apparent with patterns than individual behaviours. These associations identified girls and older children at increased risk for unhealthier lifestyle patterns and emphasise the need for tailored prevention and intervention strategies. The study’s novelty provides evidence on behavioural patterns accounting for the effects of sleep (less explored) and associations for understudied family level correlates.

Findings for an association between child sex and behavioural patterns varied by age in the current study. At 6–8 y, a clear picture of the existing evidence could not be established as only one previous study in this age group included all four behaviours and it reported no evidence of associations [21]. In contrast, girls at 6–8 y in our study were less prevalent than boys in mixed patterns (high physical activity and unhealthy eating), compared to the healthy pattern, with no associations observed for unhealthy patterns. Another study investigating three behaviours also found lower prevalence of girls in unhealthy patterns (high sedentary behaviour and energy dense food intake) compared to the healthy patterns [18]. Although the patterns across studies were not directly comparable, and the lack of available evidence for this age group precludes firm conclusions, there is some suggestion of sex differences in patterns that broadly follow what is seen for individual behaviours, in particular, girls displaying patterns comprising healthier diets than boys at 6–8 y. At 9–11 y, findings were more consistent across studies but in the opposite direction to what was observed for younger children. Similar to our findings, two studies [11,23] investigating all four behavioural domains reported a higher proportion of girls in either unhealthy or mixed patterns (lower physical activity pattern with either a poorer diet [11] or a better diet [23]) and lower prevalence in healthy patterns than boys. Other studies examining less than four behavioural domains reported higher prevalence of girls displaying patterns that are either more sedentary [31,32,33], have healthier diets [33], and have lower physical activity [31,32] compared to boys. Conversely, one study reported a higher proportion of boys displaying healthy patterns and girls displaying unhealthy patterns [34]. The associations with sex may be dependent on the number of behaviours included in the study and the number of behaviours that comprise the derived patterns. Furthermore, individual behaviours that display strong associations with sex appear to drive the associations with correlates for those patterns, where these behaviours appear to be dominant. For example, girls are shown to have healthy diets [35] and similarly displayed more healthy patterns than boys at 6–8 y where diet appeared to be the dominant behaviour. Although tracking was not assessed and most previous studies have also been cross-sectional, the differences in findings by sex at the two age groups suggest these behaviours and behavioural patterns change during this age period. As most studies examined children above 9 y, more evidence is needed for the younger age group before conclusions are drawn. However, as differences appear fairly consistent between boys and girls, sex specific prevention and intervention efforts appear warranted.

Although age as a correlate for behavioural patterns has been infrequently investigated, our findings at both time points were consistent with previous evidence [15]. At both 6–8 y and 9–11 y, our study reported increased risk of being in the unhealthy pattern with increasing age. This is corroborated by the evidence. Children displayed patterns comprising higher physical activity levels below nine years [36,37]. Children were also reported to be more sedentary with increasing age from 6 to 9 years [32]. These findings also mirror those seen for individual behaviours [38]. The findings warrant an increase in health promotion efforts with age. Most previous studies have examined associations between patterns and health outcomes at different ages, but not age associations with patterns themselves making this a novel aspect of this study.

In considering family level correlates, this study reported dissimilar evidence to previous studies for associations with parental education and marital status. We found little evidence of associations for parent education or marital status at either time point. Most previous studies reported higher parental education was associated with healthier patterns and inverse findings for lower parental education [12,15,16], including two studies with patterns including all four behavioural domains [20,23]. However, some studies that investigated only two or three behavioural domains in their patterns reported no association with parent education [33,34,39], consistent with our findings. Only one previous study, involving patterns across three behaviours, investigated marital status [40], finding an association between unmarried or non-de facto relationship parents and children displaying unhealthy patterns (high sedentary behaviour and discretionary food consumption) compared to the other patterns in their study. Given a number of novel correlates were investigated in this study (e.g., possession of health care card), it was not possible to compare their null findings with other research on four behaviour patterns in this age group.

The general lack of evidence for family level correlates in this study might suggest that, unlike child level correlates, the dominant behaviours in patterns may not drive associations for family level correlates and there may be other factors at play. These inconsistencies could be due to the greater complexity of family level correlates compared with child level correlates, and that as children grow-up, social influences are less concentrated in the family, and more in the outside world. A general paucity of evidence, combined with the complexity of different behavioural patterns across studies, makes it difficult to draw conclusions about the importance of family level correlates in influencing behavioural patterns.

To our best knowledge, this was one of few studies to include four behavioural domains (twelve behavioural variables) to derive patterns at two time points and investigate their associations with child and family level correlates. The inclusion of sleep, in addition to frequently examined behaviours (dietary intake, physical activity, and sedentary behaviour) is a strength. Exploring associations of behavioural patterns with understudied correlates including parental working hours, possession of a pension or health care card, and sibling type were also novel. Although some bias exists, as most behavioural and correlate data were captured using subjective methods, these measures have shown good reliability. Nevertheless, this study also included objectively measured physical activity and sedentary behaviour, thereby improving confidence in the patterns obtained. It is worth noting that LPA is data driven, therefore the results of the present study may not be directly comparable to other studies. Its use, however, is beneficial in understanding the synergistic influence of these behaviours not captured using *a priori* methods (pre-categorised variables based on certain criteria). The distribution of children across the patterns derived by LPA could have resulted in lower power in detecting associations with categorical family factors, therefore larger samples would be beneficial. For associations with child sex, the smaller number of boys and girls in the pattern groups could have led to less accurate estimation of relative risk ratios. The current study included a higher proportion of university educated parents than the national average [41], which may limit the generalizability of the study findings to the wider Australian population. Given the link between education and other family level correlates, the high proportion of educated parents could have impacted the variability in other correlates and hence potential to observe associations in this sample compared to one with better representation of social diversity and disadvantage. Given the patterns identified at the two time points were different, they were not comparable and precluded longitudinal investigation of associations with correlates. The cross-sectional study design limited inference of causal relationships between the correlates and behavioural patterns. Other child and family correlates could not be explored due to limitations of data availability in this cohort.

## 5. Conclusions

This study aimed to investigate cross-sectional associations between child and family level correlates and behavioural patterns (derived using four behavioural domains), in a cohort of Australian children aged 6–8 y and 9–11 y. At both time points, child sex and age were cross-sectionally associated with behavioural patterns. Of the family level correlates investigated, only parent working hours showed evidence of associations at 9–11 y. Little evidence of associations was seen for other correlates investigated. The findings from the study are significant to inform prevention and intervention efforts targeting sub-groups, specifically girls and older children, at higher risk of unhealthy behavioural patterns. Overall findings suggest that child level correlates might be more important to consider over family level correlates, given consistent evidence of associations across studies examining behaviours individually and as patterns. Further studies to increase the evidence base and exploring other correlates are warranted with inclusion of all four behavioural domains (dietary intake, physical activity, sedentary behaviour, and sleep) desirable.

## Figures and Tables

**Table 1:** Characteristics of the cohort at 6–8 y and 9–11 y.

Variable	6–8 y (n = 335)	9–11 y (n = 339)
Child Age (Years) [Mean ± SD (Range)]	7.5 ± 0.7 (6.0–9.1)	10.6 ± 0.7 (9.0–12.2)
Sex [n (%)]		
Male	191 (57.0)	190 (56.1)
Female	144 (43.0)	149 (43.9)
Parent Education [n (%)]		
Below university level	109 (32.5)	105 (31.0)
University and above	226 (67.5)	234 (69.0)
Marital status [n (%)]		
Married/de facto	306 (91.3)	301 (88.8)
Other	29 (8.7)	38 (11.2)
Number of working hours [Mean ± SD (range)]	4.7 ± 3.9 (0–19.2)	5.3 ± 3.6 (0–16)
Pension or Health care card		
Yes	35 (10.4)	37 (10.9)
No	300 (89.6)	302 (89.1)
Sibling type [n (%)]		
Only child	43 (12.8)	41 (12.1)
Oldest child	134 (40.0)	140 (41.3)
Youngest child	113 (33.7)	109 (32.2)
Middle child	45 (13.5)	49 (14.4)

Abbreviations: MVPA = moderate- to vigorous-intensity physical activity; SD = standard deviation.

**Table 2:** Associations of behavioural patterns and child and family level correlates at 6–8 y (n = 335).

Correlates	Bivariate Model ^				Multivariate Model ^			
	Unhealthy (n = 69)		Active Unhealthy Eaters (n = 74)		Unhealthy (n = 69)		Active Unhealthy Eaters (n = 74)	
	RRR (95% CI)	*p*-Value	RRR (95% CI)	*p*-Value	RRR (95% CI)	*p*-Value	RRR (95% CI)	*p*-Value
**Child sex (ref: male)**								
** Female**	1.34 (0.75, 2.39)	0.324	0.37 (0.22, 0.63)	0.000	1.56 (0.83, 2.93)	0.170	0.34 (0.19, 0.59)	0.000
**Child age (years)**	1.89 (1.18, 3.02)	**0.008**	0.51 (0.33, 0.77)	**0.002**	2.00 (1.22, 3.27)	**0.006**	0.47 (0.30, 0.74)	**0.001**
**Below university parent education (ref)**					-	-	-	-
** University and above**	0.68 (0.36, 1.30)	0.243	0.72 (0.39, 1.31)	0.280				
**Married/de facto (ref)**								
** Other**	2.16 (0.76, 6.17)	0.151	2.58 (0.88, 7.51)	**0.084**	2.14 (0.58, 7.91)	0.253	1.11 (0.28, 4.44)	0.885
**Working hours**	1.01 (0.94, 1.09)	0.756	1.01 (0.95, 1.08)	0.778	-		-	-
**Health care or Pension card (ref: no)**								
** Yes**	1.91 (0.75, 4.87)	0.177	2.46 (0.97, 6.27)	**0.059**	1.20 (0.37, 3.91)	0.758	2.41 (0.71, 8.17)	0.157
**Sibling type (ref: only child)**		0.320		0.102	-	-	-	-
** Oldest child**	0.67 (0.28, 1.59)		0.55 (0.22, 1.38)					
** Youngest child**	1.08 (0.47, 2.47)		1.17 (0.41, 3.35)					
** Middle child**	1.38 (0.54, 3.56)		0.70 (0.21, 2.28)					

Abbreviations: RRR = relative risk ratio; CI = confidence interval; ref = reference. ^ Reference group for the outcome is the non-sedentary healthy eaters pattern (n = 192). Note: *p*-values in bold indicate statistically significant associations.

**Table 3:** Associations of behavioural patterns and child and family level correlates at 9–11 y (n = 339).

Correlates	Bivariate Model ^				Multivariate Model ^			
	Unhealthy (n = 63)		Intermediate (n = 218)		Unhealthy (n = 63)		Intermediate (n = 218)	
	RRR (95% CI)	*p*-Value	RRR (95% CI)	*p*-Value	RRR (95% CI)	*p*-Value	RRR (95% CI)	*p*-Value
**Child sex (ref: male)**								
** Female**	11.07 (4.62, 26.53)	**0.000**	6.65 (2.93, 15.06)	**0.000**	11.63 (4.71, 28.72)	**0.000**	6.57 (2.84, 15.24)	**0.000**
**Child age (years)**	1.87 (1.17, 2.99)	**0.009**	1.09 (0.76, 1.55)	0.652	2.07 (1.26, 3.39)	**0.004**	1.16 (0.80, 1.70)	0.434
**Below university parent education (ref)**								
** University and above**	0.74 (0.30, 1.79)	0.500	0.65 (0.32, 1.28)	0.211	-	-	-	-
**Married/defacto (ref)**								
** Other**	0.78 (0.25, 2.44)	0.670	0.74 (0.28, 1.92)	0.532	-	-	-	-
**Working hours**	0.91 (0.81, 1.01)	0.084	0.90 (0.83, 0.98)	**0.013**	0.92 (0.82, 1.03)	0.159	0.91 (0.83, 0.99)	**0.027**
**Health care card/Pension** **(ref: no)**								
** Yes**	1.08 (0.32, 3.72)	0.899	1.07 (0.38, 3.01)	0.895	-	-	-	-
**Sibling type** **(ref: only child)**					-	-	-	-
** Oldest child**	0.81 (0.30, 2.19)	0.363	0.83 (0.31, 2.26)	0.490				
** Youngest child**	0.59 (0.21, 1.67)		0.66 (0.25, 1.72)					
** Middle child**	1.65 (0.42, 6.42)		1.47 (0.38, 5.64)					

Abbreviations: RRR = relative risk ratio, CI = confidence interval, ref = reference. ^ Reference group is the active and non-sedentary pattern (n = 58). Note: *p*-values in bold indicate statistically significant associations.

## Data Availability

Ethical restrictions related to the consent given by participants at the time of study commencement limit the datasets analysed for the current study being publicly available. An ethically compliant dataset may be made available by the corresponding author on reasonable request and upon approval by the Deakin University Human Research Ethics Committee.

## Supplementary Materials

The following are available online at https://www.mdpi.com/article/10.3390/children8111023/s1, Table S1: Pattern characteristics using LPA at T2 (6–8 years) and T3 (9–11 years).
