# Supplementary material for: Associations between Child and Family Level Correlates and Behavioural Patterns in School-Aged Children
Source: Children (Basel). 2021 Nov 7;8(11):1023. doi: 10.3390/children8111023 (PMC8620248; doi:10.3390/children8111023)
Supplement: Supplementary file 1 [file children-08-01023-s001.zip › children-1407530-supplementary.pdf]

Table S1: Pattern characteristics using LPA at T2 (6-8 years) and T3 (9-11 years).

	Behaviours	T2 [Mean (SD)]			T3 [Mean (SD)]		
		Unhealthy (n=69)	Non-sedentary healthy eaters (n=192)	Active unhealthy eaters (n=74)	Unhealthy (n=63)	Intermediate (n=218)	Active and non-sedentary (n=58)
Diet	Fruit intake	**1.5 (0.9)** [a]	**2.7 (1.3)** [a]	**2.0 (1.0)** [a]	**1.9 (1.2)** [ab]	**2.8 (1.2)** [a]	**2.7 (1.1)** [b]
	Vegetable intake	**2.1 (1.2)** [ab]	**3.2 (1.3)** [a]	**2.9 (1.1)** [b]	3.0 (1.5)	3.5 (1.2)	3.5 (1.2)
	Sweet discretionary food intake	**1.8 (0.7)** [a]	**1.4 (0.6)** [ab]	**1.7 (0.7)** [b]	1.5 (0.8)	1.5 (0.8)	1.6 (0.7)
	Savoury discretionary food intake	**1.2 (0.5)** [a]	**1.1 (0.5)** [b]	**1.4 (0.4)** [ab]	**1.1 (0.5)** [a]	**1.1 (0.5)** [b]	**1.2 (0.5)** [ab]
PA	Organised sport	**17.8 (16.7)** [a]	**26.0 (22.0)** [ab]	**21.7 (15.4)** [b]	**17.1 (18.9)** [ab]	**36.0 (27.0)** [a]	**54.9 (33.2)** [b]
	Outdoor play	**113.4 (56.2)** [ab]	**145.4 (77.9)** [a]	**161.4 (74.1)** [b]	**99.2 (62.5)** [ab]	**153.8 (82.7)** [a]	**182.5 (88.4)** [b]
	MVPA	**75.7 (13.8)** [a]	**106.5 (15.8)** [a]	**146.8 (19.8)** [a]	**32.8 (10.8)** [a]	**59.6 (14.2)** [a]	**100.6 (16.1)** [a]
SB	Screen time	**126.0 (68.4)** [a]	**79.3 (51.9)** [ab]	**99.1 (45.3)** [b]	**150.3 (108.4)** [ab]	**102.7 (59.0)** [a]	**103.1 (61.1)** [b]
	Videogame time	32.3 (43.3)	22.0 (29.7)	19.0 (25.1)	**56.6 (92.7)** [a]	**13.2 (23.9)** [a]	22.3 (44.5)
	Quiet play time	65.5 (57.6)	50.9 (36.6)	50.4 (32.5)	48.5 (29.9)	57.3 (38.9)	46.8 (28.1)
	Sedentary time	**404.3 (30.9)** [a]	**363.1 (25.9)** [a]	**304.5 (24.8)** [a]	**503.9 (35.0)** [a]	**439.8 (30.5)** [a]	**374.4 (31.3)** [a]
Sleep	Sleep	**606.9 (40.4)** [ab]	**631.9 (47.6)** [a]	**629.7 (44.6)** [b]	**556.0 (52.8)** [ab]	**597.3 (50.0)** [a]	**592.5 (44.9)** [b]

Abbreviations: LPA; latent profile analysis; T2; wave two; T3; wave three
All dietary intakes are all reported in times/day (frequency of consumption) and activity measures are all in mins/day.
Values in bold indicate statistically significant pairwise comparisons with same superscripts indicating significant differences between those pattern groups.
